# Supplementary material for: Ready student one: Exploring the predictors of student learning in virtual reality
Source: PLoS One. 2020 Mar 25;15(3):e0229788. doi: 10.1371/journal.pone.0229788 (PMC7094822; doi:10.1371/journal.pone.0229788)
Supplement: S1 Table — Supporting data for Fig 6. (PDF) [file pone.0229788.s001.pdf]

## Supporting information

**S1 Table Modeling interactions between video game experience and gender with condition** Supporting data for Fig. 6

| Video game experience | | | | | | | |
|---|---|---|---|---|---|---|---|
| Condition | Exp. | Intercept | SE | p | Slope | SE | p |
| VR | Yes | 4.94 | 0.50 | 3.0E-11 | **4.12** | 0.71 | 1.8E-06 |
| VR | No | 4.62 | 0.37 | 7.1E-20 | **2.90** | 0.53 | 5.1E-07 |
| Desktop | Yes | 4.48 | 0.56 | 7.7E-10 | 3.00 | 0.79 | 4.9E-04 |
| Desktop | No | 3.89 | 0.39 | 5.5E-15 | 3.42 | 0.55 | 4.0E-08 |
| Hands-on | Yes | **5.11** | 0.48 | 5.6E-15 | 3.46 | 0.67 | 3.9E-06 |
| Hands-on | No | **3.68** | 0.41 | 1.4E-12 | 3.23 | 0.58 | 7.6E-07 |
| Gender | | | | | | | |
| Condition | Gender | Intercept | SE | p | Slope | SE | p |
| VR | Male | 4.90 | 0.61 | 2.4E-07 | **4.70** | 0.86 | 3.7E-05 |
| VR | Female | 4.67 | 0.35 | 5.2E-23 | **2.96** | 0.49 | 4.1E-08 |
| Desktop | Male | 3.86 | 1.07 | 3.7E-03 | 4.14 | 1.52 | 1.8E-02 |
| Desktop | Female | 4.08 | 0.34 | 4.2E-21 | 3.12 | 0.48 | 2.6E-09 |
| Hands-on | Male | 4.86 | 0.82 | 2.8E-06 | 2.50 | 1.16 | 4.0E-02 |
| Hands-on | Female | 4.18 | 0.36 | 1.6E-19 | 3.61 | 0.50 | 2.5E-10 |
