# Supplementary material for: Ready student one: Exploring the predictors of student learning in virtual reality
Source: PLoS One. 2020 Mar 25;15(3):e0229788. doi: 10.1371/journal.pone.0229788 (PMC7094822; doi:10.1371/journal.pone.0229788)
Supplement: S2 Table — Supporting data for Fig 7. (PDF) [file pone.0229788.s002.pdf]

**S2 Table  Modeling interactions between academic major, and VR experience quantity with condition** Supporting data for Fig. 7

| Academic Major | | | | | | | |
|---|---|---|---|---|---|---|---|
| Condition | Major | Intercept | SE | p | Slope | SE | p |
| VR | Science | 5.40 | 0.62 | 1.6E-09 | 3.27 | 0.87 | 8.1E-04 |
| VR | Non-Science | 5.43 | 0.99 | 1.5E-04 | 2.29 | 1.41 | 1.3E-01 |
| Desktop | Science | 4.38 | 0.37 | 1.2E-08 | **3.88** | 0.52 | 3.4E-06 |
| Desktop | Non-Science | 4.17 | 0.79 | 2.7E-05 | **2.17** | 1.12 | 6.5E-02 |
| Hands-on | Science | **5.75** | 0.89 | 1.7E-06 | 2.83 | 1.26 | 3.5E-02 |
| Hands-on | Non-Science | **4.00** | 0.72 | 1.3E-04 | 3.00 | 1.02 | 1.3E-02 |

| Quantity of VR experience | | | | | | | |
|---|---|---|---|---|---|---|---|
| Condition | Quantity | Intercept | SE | p | Slope | SE | p |
| VR | None | **5.41** | 0.52 | 2.8E-13 | 2.95 | 0.73 | 2.2E-04 |
| VR | Little | 4.29 | 0.47 | 2.4E-11 | 3.43 | 0.66 | 6.8E-06 |
| VR | Moderate/A lot | 4.23 | 0.63 | 6.3E-07 | 3.54 | 0.89 | 5.8E-04 |
| Desktop | None | 4.14 | 0.49 | 2.1E-10 | 3.00 | 0.70 | 1.0E-04 |
| Desktop | Little | 4.28 | 0.48 | 3.5E-12 | 3.72 | 0.69 | 1.3E-06 |
| Desktop | Moderate/A lot | 3.29 | 0.55 | 6.2E-05 | **2.14** | 0.77 | **1.7E-02** |
| Hands-on | None | 4.85 | 0.65 | 5.2E-09 | 2.95 | 0.91 | 2.6E-03 |
| Hands-on | Little | 4.17 | 0.41 | 1.7E-14 | 3.90 | 0.58 | 8.3E-09 |
| Hands-on | Moderate/A lot | 3.89 | 0.79 | 1.6E-04 | 2.33 | 1.12 | 5.4E-02 |
